# The Role of Artificial Intelligence in Exercise-Based Cardiovascular Health Interventions: A Scoping Review

**DOI:** 10.3390/jfmk10040409

**Published:** 2025-10-21

**Authors:** Asterios Deligiannis, Panagiota Sotiriou, Pantazis Deligiannis, Evangelia Kouidi

**Affiliations:** 1 Sport Medicine Laboratory, School of Physical Education and Sport Science, Aristotle University, 57001 Thessaloniki, Greece; nayiasot@gmail.com (P.S.); ekouidi@phed.auth.gr (E.K.); 2 Microsoft Research, 14820 NE 36th St, Redmond, WA 98052, USA; pdeligia@me.com

**Keywords:** artificial intelligence, cardiovascular diseases, exercise, precision medicine, cardiac rehabilitation, digital health

## Abstract

**Background:** As cardiovascular medicine advances rapidly, the integration of artificial intelligence (AI) has garnered increasing attention. Although AI has been widely adopted in diagnostics, risk prediction, and decision support, its application in exercise-based cardiovascular rehabilitation is still limited, representing a new and promising research frontier. **Objective:** This scoping review aimed to identify and analyze original studies that have applied AI to exercise-based interventions designed to improve cardiovascular outcomes. **Methods:** Following the PRISMA-ScR guidelines, PubMed, Scopus, Web of Science, Embase, and IEEE Xplore were searched for articles published between January 2015 and August 2025. Eligible studies were peer-reviewed by human research employing AI (machine learning or deep learning) to deliver, adapt, or monitor an exercise intervention with cardiovascular outcomes. Reviews, diagnostic-only studies, protocols without data, and animal studies were excluded. Non-original works (reviews, protocols), animal studies, and purely diagnostic applications were excluded, ensuring a strict focus on AI applied within exercise interventions. Data extraction focused on study design, AI method, exercise modality, outcomes, and findings. **Results:** From 2183 records, nine studies met the inclusion criteria (two RCTs, feasibility pilots, and validation studies). Designs included feasibility pilots, randomized controlled trials (RCTs), and validation studies. AI applications encompassed adaptive step goals, reinforcement learning for engagement, coaching apps, machine learning–based exercise prescription, and continuous monitoring (e.g., VO_2_ estimation). These AI methods, such as machine learning and reinforcement learning, were used to personalize exercise interventions and improve cardiovascular outcomes. Reported outcomes included blood pressure reduction, improved adherence, increased daily steps, improvement in VO_2_max, continuous physiological monitoring, and enhanced diagnostic accuracy. **Conclusions:** Findings demonstrate that AI has the potential to significantly enhance cardiovascular rehabilitation. It can personalize exercise prescriptions, enhance adherence, and facilitate safe monitoring in home settings. However, the evidence base remains preliminary, with very few RCTs and substantial methodological heterogeneity. Future research must prioritize large-scale clinical trials, explainable AI, and equitable implementation strategies to ensure clinical translation.

## 1. Introduction

Cardiovascular disease (CVD) continues to represent the leading cause of mortality and morbidity worldwide, accounting for approximately 17.9 million deaths annually and placing immense strain on health systems [1]. Structured physical activity and supervised exercise programs are consistently recognised as cornerstones for both prevention and secondary rehabilitation [2]. Regular participation in exercise not only improves aerobic capacity and reduces major risk factors, such as hypertension, but also lowers hospital readmission rates and enhances overall cardiovascular prognosis [2].Nevertheless, implementation in real-world settings faces considerable challenges, including variability in individual response to training, restricted availability of personalised interventions, and difficulties in sustaining long-term adherence.

Artificial intelligence (AI), particularly machine learning (ML) and deep learning (DL), has rapidly become a disruptive force in medicine [3,4,5]. Regular participation in exercise not only improves aerobic capacity and reduces major risk factors, such as hypertension, but also lowers hospital readmission rates and enhances overall cardiovascular prognosis.

While its earliest applications were concentrated in diagnostic imaging and risk stratification [4,5] AI is now extending into therapeutic and preventive domains, including cardiovascular rehabilitation [6,7]. A recent comprehensive overview also highlights its integration into medical decision-making and diagnostics, underscoring the need for explainability and trustworthiness [8]. Its capacity to analyse large, complex datasets and to generate adaptive, data-driven recommendations makes it especially suited for exercise interventions, which require personalisation, continuous feedback, and scalability.

Several domains highlight how artificial intelligence may transform the design and delivery of exercise-based cardiovascular interventions. Perhaps the most widely discussed contribution is the capacity of AI to personalize exercise prescriptions based on individual physiological data, comorbidities, and response patterns [9,10].Instead of relying on standardized exercise protocols, machine learning algorithms are capable of incorporating information about an individual’s baseline fitness level, comorbidities, and physiological responses to previous sessions to generate more tailored programs [11]. This level of personalization is clinically meaningful, as it minimizes the risks of undertraining or overexertion and promotes safety and effectiveness in rehabilitation and prevention settings [9]. Such personalization is clinically important because it minimises the risks of undertraining or overexertion encuring that each participant receives a regimen optimised for both safety and effectiveness.

Another critical area is continuous monitoring. Wearable devices enhanced with AI now make it possible to track vital parameters such as heart rate, oxygen consumption, and movement patterns in real time [7,8]. The integration of these data streams into adaptive models allows exercise intensity to be dynamically adjusted as conditions change, bridging the gap between supervised clinical sessions and unsupervised activity in daily life [11].

Equally important is promoting adherence, a longstanding challenge in cardiovascular rehabilitation. Reinforcement learning algorithms and adaptive digital platforms have demonstrated the ability to deliver motivational prompts that evolve according to patient behaviour [6]. By tailoring feedback to the individual’s level of engagement, mood, or activity pattern, these systems maintain interest and encourage consistent participation over time. By tailoring feedback to patient engagement, mood, and behavioral responses, these AI systems sustain long-term adherence, one of the most persistent barriers to effective rehabilitation [6,7].This approach addresses one of the most persistent limitations of traditional rehabilitation programs, which often have high dropout rates after the initial months.

Finally, the scalability of AI-enabled interventions holds significant promise. Because these systems can automate elements of coaching, monitoring, and data analysis, they enable delivery of personalized rehabilitation services to much larger populations than would be feasible with clinician-led models alone [12]. This is particularly relevant in low-resource settings, where specialist centres and trained staff are scarce.

Early exploratory studies first demonstrated that artificial intelligence could be meaningfully integrated into exercise-based cardiovascular care. Kwon et al. [6] applied machine learning algorithms to wearable-derived physiological data, showing that exercise prescriptions could be personalized rather than standardized. Around the same period, Piwek et al. [7] examined early wearable technologies, highlighting both their potential for real-time monitoring and the challenges of adherence and data accuracy. Building upon these early proofs of concept, Zhou et al. [8] conducted one of the first randomized controlled trials using adaptive, AI-generated step goals, while Aguilera et al. [9] applied reinforcement learning to behavioral interventions, demonstrating enhanced long-term engagement.

Subsequent investigations expanded the scope of AI applications in cardiovascular health. Meder et al. [10] emphasized population-level prevention strategies enabled by AI, whereas Kufel et al. [11] explored its diagnostic potential in cardiovascular assessment. Topol [12] further contextualized these advances, describing how AI augments, rather than replaces, human clinical expertise in modern precision medicine. Together, these foundational studies illustrate the progressive evolution of AI—from early feasibility demonstrations to clinically relevant, scalable applications—and provide the scientific rationale for the present review, which aims to map and synthesize this emerging field systematically.

Therefore, the purpose of this review is to: (1) map the available studies that have applied AI in exercise-based cardiovascular interventions between 2015 and 2025; (2) examine their methodological approaches, study populations, and outcomes; and (3) identify achievements, limitations, and future priorities. Through this synthesis, the review aims to provide a clearer understanding of the potential for AI to enhance exercise-based cardiovascular care and outline the steps required for its safe and equitable translation into clinical practice.

## 2. Methods

### 2.1. Study Protocol

This scoping review was designed and conducted in accordance with the PRISMA-ScR (Preferred Reporting Items for Systematic Reviews and Meta-Analyses extension for Scoping Reviews) guidelines [13]. The use of the PRISMA-ScR framework provided a transparent, systematic, and reproducible structure. By adhering to these guidelines, the review process aimed to minimise potential sources of bias and to provide a comprehensive and reliable overview of the existing literature on the integration of artificial intelligence in exercise-based cardiovascular interventions. The review protocol was prospectively published on Preprints.org, enhancing transparency. A completed PRISMA-ScR checklist and the corresponding PRISMA flow diagram are provided in the Supplementary Materials.

The Population–Concept–Context (PCC) model guided the review framework, which is widely recommended for scoping reviews. This model facilitated a structured and transparent selection process by focusing on (1) human participants engaged in exercise-based interventions relevant to cardiovascular health, (2) the application of artificial intelligence methods—specifically machine learning and deep learning—to deliver, adapt, or monitor such interventions, and (3) healthcare or community-based settings, including clinical rehabilitation and preventive programs. Applying the PCC framework ensured consistent eligibility criteria and comparability across heterogeneous study designs.

### 2.2. Eligibility CriteriaTT

To ensure that the review captured only relevant and high-quality studies, specific inclusion and exclusion criteria were applied. Eligible studies were restricted to peer-reviewed, original human research that applied artificial intelligence methods—specifically machine learning or deep learning—to the design, delivery, adaptation, or monitoring of exercise interventions with measurable cardiovascular outcomes. Such outcomes included, but were not limited to, blood pressure, heart rate, oxygen consumption (VO_2_), VO_2_max, exercise adherence, and diagnostic accuracy in exercise-related contexts.

Studies were excluded if they involved only animal models or laboratory-based simulations without human data, as these do not directly translate into clinical or rehabilitation settings. Similarly, investigations that employed AI solely for diagnostic purposes without incorporating an exercise intervention were not considered. Non–original works, such as reviews, editorials, commentaries, or study protocols lacking empirical data, were also excluded. These criteria ensured that the review remained focused on original, human-based research that demonstrated practical applications of AI within exercise-based cardiovascular health.

### 2.3. Information Sources and Search Strategy

A systematic search strategy was developed to identify all relevant studies published between January 2015 and August 2025. Five major electronic databases were selected: PubMed, Scopus, Web of Science, Embase, and IEEE Xplore. These databases were chosen for their comprehensive coverage of biomedical, health sciences, and engineering literature, ensuring a multidisciplinary approach to the topic.

The search combined both free-text terms and controlled vocabulary, including Medical Subject Headings (MeSH) and equivalent indexing terms. Keywords such as “artificial intelligence,” “machine learning,” “deep learning,” “exercise,” “physical activity,” and “cardiovascular health” were systematically combined using Boolean operators. Filters were applied to restrict results to human studies and peer-reviewed publications. No language restrictions were applied, although all retrieved studies that met the eligibility criteria were published in English. Detailed full search strings for each database are provided in Supplementary Table S1.

### 2.4. Study Selection

The study selection process followed a two-stage screening approach. First, titles and abstracts of all retrieved records were independently reviewed by two researchers. Studies that clearly did not meet the eligibility criteria were excluded at this stage. In the second stage, the full texts of potentially relevant articles were retrieved and thoroughly assessed in detail against the inclusion and exclusion criteria.

Any disagreements between the reviewers regarding eligibility were resolved through discussion until consensus was reached. Inter-reviewer agreement was calculated (>90%). When necessary, a third reviewer was consulted to adjudicate. Duplicate publications were identified and removed prior to the screening process. This rigorous and transparent approach ensured that only studies meeting the predetermined criteria were included in the final synthesis.

### 2.5. Data Extraction

Data were systematically extracted from each included study using a structured charting form. Extracted variables included the name of the first author, year of publication, country where the study was conducted, and the study design (e.g., randomized controlled trial, pilot study, feasibility study, validation trial). Participant characteristics such as sample size, age, sex distribution, and comorbidities were systematically recorded.

Additionally, the type of AI methodology applied was identified, distinguishing between machine learning, deep learning, reinforcement learning, and generative models. Exercise modality (intervention type, duration, frequency, intensity) and cardiovascular outcomes (blood pressure, VO_2_, VO_2_max, adherence, diagnostic accuracy) were captured.

### 2.6. Data Synthesis

Given the methodological diversity of the included studies, data were synthesised descriptively rather than quantitatively. Randomised controlled trials were reported in detail with respect to intervention design, comparators, and measured outcomes.

Pilot and feasibility studies were synthesized narratively with a focus on feasibility, acceptability, and proof-of-concept results. Results were grouped thematically by AI application domain (adaptive goal setting, digital coaching, wearable monitoring, exercise prescription engines, diagnostic augmentation, population-level approaches).

### 2.7. Statistics

Given the exploratory nature of this scoping review and the methodological heterogeneity of the included studies, no meta-analysis was performed. Instead, data were summarised descriptively.

Continuous outcomes (e.g., blood pressure, VO_2_, VO_2_max) were described with mean differences or effect sizes when available. Diagnostic performance metrics such as AUC were reported as provided by authors. This descriptive statistical approach is consistent with PRISMA-ScR guidelines [13] and was deemed most appropriate given the limited number of studies, their small sample sizes, and variability in intervention designs.

## 3. Results

### 3.1. Study Selection

The initial database search identified a total of 2183 records published between January 2015 and August 2025. Following the removal of duplicate entries, 2000 unique records were retained for the screening process. Title and abstract screening served as the first stage of evaluation. During this step, the majority of articles were excluded because they clearly did not meet the predefined eligibility criteria. At this stage, most exclusions were due to studies focusing on diagnostic-only AI applications without exercise interventions, investigations limited to animal models, and non-original works such as reviews, commentaries, or theoretical discussions. 

After this preliminary screening, 40 articles were considered sufficiently relevant to warrant full-text review. At this stage, each study was carefully examined against the inclusion and exclusion criteria outlined in the methods section. Particular attention was paid to whether AI methods were directly applied to exercise-based interventions and whether cardiovascular outcomes were reported. Studies without an explicit AI component, or reporting only usability outcomes without cardiovascular endpoints, were excluded. Protocols that proposed but had not yet implemented interventions were also omitted.Similarly, protocols that proposed but had not yet implemented AI-driven interventions were not included, as the focus of this review was on empirical evidence rather than planned or conceptual projects.

Of the 40 articles reviewed in full, 29 were excluded for reasons such as lack of cardiovascular outcome reporting, reliance on non-AI digital technologies, absence of exercise-based components, or insufficient methodological detail to assess the role of AI. Ultimately, 9 studies fulfilled all inclusion criteria and were selected for detailed synthesis in this review. These included two randomised controlled trials, several feasibility and pilot studies, and more recent validation or single-arm interventional trials.

**Figure 1:**
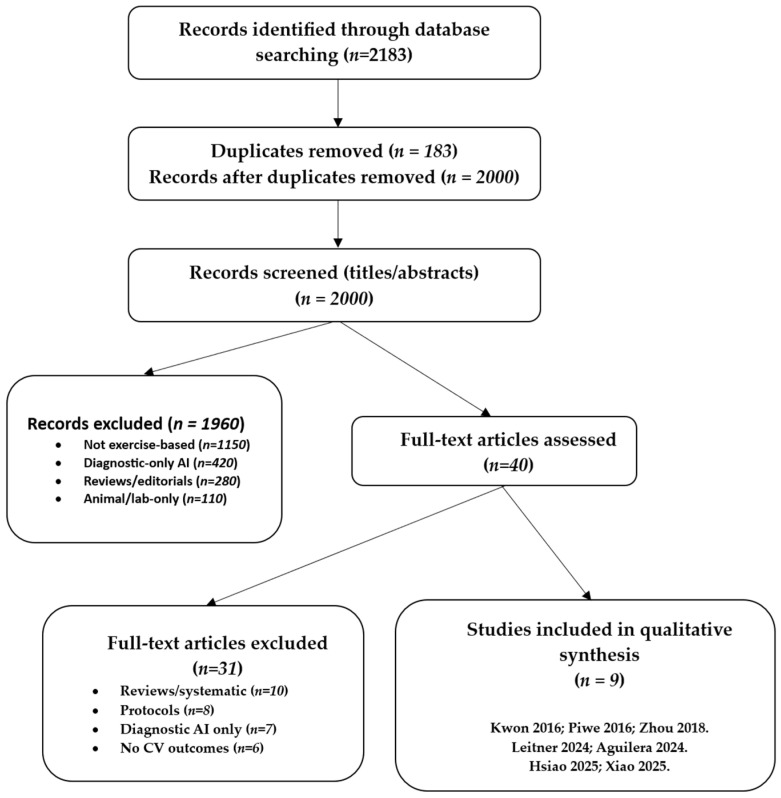
Flowchart of the study.PRISMA-ScR flow diagram of study selection (January 2015–August 2025). After screening, *n* = 9 studies were included [6,7,8,9,10,14,15,16,17]. Full search strings are provided in Table S1. The process followed PRISMA-ScR [13].

The overall process of identification, screening, eligibility assessment, and inclusion followed the PRISMA-ScR framework [13]. The flow of study selection is summarised in Figure 1, which visually depicts the number of records identified, screened, reviewed in full, excluded, and finally included in the analysis. Details are provided in Supplementary Table S2. This structured approach ensured transparency and reproducibility in the selection of studies, while also providing a clear rationale for excluding records that did not meet the eligibility thresholds.

### 3.2. Characteristics of Included Studies

The earliest contributions were feasibility or pilot studies [6,7], typically involving small participant groups and short follow-up periods, focusing primarily on establishing technical feasibility.These investigations were typically conducted with relatively small participant groups and short follow-up periods, focusing primarily on establishing the technical feasibility of integrating AI methods into exercise-based cardiovascular interventions. Although limited in scope, they provided important groundwork for later, more rigorous studies by demonstrating that AI-enabled tools could be effectively applied in real-world contexts.

Randomized controlled trials (RCTs) represented a smaller but highly influential portion of the evidence base. Two studies, those of Zhou et al. [8] and Aguilera et al. [9], utilized adaptive AI systems in behavioural interventions aimed at promoting physical activity. These trials enrolled larger and more diverse populations compared with pilot studies, and both demonstrated clear improvements in adherence and activity outcomes relative to traditional static prescriptions. The inclusion of RCTs within this body of literature is significant, as they offer the most substantial evidence to date that AI can enhance exercise interventions not only in terms of feasibility but also in clinical efficacy.

More recent contributions have expanded into validation and single-arm interventional designs [14,15,16,17]. These studies tended to enrol moderately sized populations and evaluated more sophisticated AI applications, such as autonomous digital coaches, neural network–driven exercise prescriptions, and advanced wearable monitoring systems. For example, Leitner et al. [16] focused on individuals with hypertension and tested an AI-powered digital health coach, while Hsiao et al. [17] validated a wearable device capable of real-time VO_2_ estimation. Xiao et al. [15] examined older adults and applied neural networks to generate individualised prescriptions. These studies reflect a trend toward testing AI applications in more clinically meaningful scenarios, where physiological outcomes, such as blood pressure reduction or improvements in aerobic capacity, are systematically measured.

The populations included across the 9 studies were also heterogeneous. Some trials recruited patients actively participating in cardiac rehabilitation programs, while others targeted individuals with chronic conditions such as diabetes, hypertension, or depression. Additional studies focused on older adults at risk of cardiovascular decline, thereby expanding the potential reach of AI-enabled interventions beyond secondary prevention to include primary prevention and geriatric populations. The variety of participant groups underscores the versatility of AI applications but also highlights challenges in comparability, as different baseline risks and clinical goals complicate synthesis across studies.

Finally, geographical distribution was skewed toward technologically advanced healthcare settings, particularly in North America, Europe, and East Asia. This reflects both the concentration of AI research infrastructure in these regions and the reliance of most interventions on digital devices such as smartphones, wearable sensors, and internet connectivity. While these contexts provide ideal testbeds for innovation, they also raise questions about the applicability of findings to resource-limited environments, where infrastructure and access remain significant barriers In summary, the characteristics of the included studies reveal a research field that is still in its formative stages but is marked by innovation and diversity. Designs ranged from small-scale feasibility pilots to RCTs and validation studies, populations spanned both clinical and community settings, and interventions addressed a spectrum of outcomes from adherence to physiological performance and diagnostic accuracy. This heterogeneity underscores both the promise and the challenges of synthesising the evidence base for AI in exercise-based cardiovascular care.

### 3.3. AI Applications in Exercise-Based Interventions

The included studies demonstrated a variety of artificial intelligence applications, covering adaptive goal setting, digital coaching, wearable-based monitoring, prescription engines, and diagnostic augmentation, thus reflecting the breadth of available technologies and evolving priorities in cardiovascular rehabilitation. Adaptive goal setting emerged as a primary application: Zhou et al. [8] tested a machine learning–based system that dynamically adjusted daily step goals according to participant activity levels, keeping them challenging yet achievable. Similarly, Aguilera et al. [9] applied reinforcement learning to personalize motivational text messages, adapting content and frequency according to user engagement, which enhanced long-term adherence.Together, these studies highlight how AI can replace static prescriptions with adaptive interventions that are more responsive to individual behaviours.

Digital coaching represented another primary domain of application. Leitner et al. [16] deployed an autonomous AI health coach designed to deliver lifestyle recommendations tailored to blood pressure readings and activity data. This digital intervention not only reduced blood pressure but also demonstrated that AI could maintain high levels of engagement without continuous clinician input, pointing to its scalability and clinical potential.

Wearable-based monitoring was also widely explored. Piwek et al. [7] investigated early consumer-grade wearable devices, recognising both their promise for tracking daily activity and the challenges related to sustained user adherence and data accuracy. More advanced wearable-based monitoring approaches were reported. For example, Hsiao et al. [17] validated an AI-driven multispectral photoplethysmography system capable of estimating VO_2_ in real time, providing a tool for adjusting intensity during unsupervised rehabilitation.

Additionally, some studies focused on exercise prescription engines. Kwon et al. [6] developed a machine learning–based platform that can tailor exercise recommendations to individual physiological responses. Xiao et al. [15] extended this work by applying a back-propagation neural network to generate personalised exercise prescriptions for older adults, ultimately demonstrating improvements in aerobic performance. These contributions illustrate the potential of AI to refine and optimise the core component of rehabilitation: the exercise prescription itself.

Diagnostic applications were also reported. For example, Liang et al. [14] used deep learning to analyze exercise stress test data, achieving higher accuracy in detecting significant coronary artery disease, linking diagnostic precision with rehabilitation planning.This application not only enhances diagnostic precision but also strengthens the link between exercise testing and rehabilitation planning, suggesting a broader integration of AI into the cardiovascular care continuum.

Beyond individual interventions Meder et al. [10] emphasized population-level applications, including prevention strategies and scaling rehabilitation programs across health systems.

### 3.4. Reported Outcomes

Reported outcomes varied widely, demonstrating multiple ways in which AI contributes to cardiovascular rehabilitation Details are provided in Supplementary Table S3. Improvements in physical activity and adherence were among the most consistently reported findings. Zhou et al. [8] demonstrated that machine learning–generated step goals significantly increased daily step counts compared with static prescriptions, while Aguilera et al. [9] showed that reinforcement learning–driven text messaging improved adherence and sustained activity in individuals with diabetes and depression. These findings suggest that AI-based personalisation can help overcome one of the most persistent barriers in lifestyle interventions: maintaining long-term engagement.

Positive cardiovascular outcomes were also observed. Leitner et al. [16] reported clinically meaningful reductions in both systolic and diastolic blood pressure among participants using an autonomous AI health coach. This highlights the potential of AI not only to encourage behaviour change but also to directly improve clinical risk factors associated with cardiovascular morbidity and mortality. Similarly, Xiao et al. [15] documented significant gains in VO_2_max and overall exercise capacity in older adults following neural network–driven individualised prescriptions, demonstrating that AI can enhance cardiorespiratory fitness in populations where functional decline is a pressing concern.

Continuous monitoring outcomes were validated by Hsiao et al. [17], who showed that AI-based photoplethysmography could reliably estimate VO_2_ during unsupervised rehabilitation. This capability enables the real-time adjustment of exercise intensity, thereby enhancing patient safety outside clinical environments. Kwon et al. [6] further confirmed that machine learning–guided prescriptions could improve HR/VO_2_ matching, increasing both the precision and adherence of prescribed training sessions.

In the diagnostic sphere, Liang et al. [14] reported that their deep learning model achieved an area under the curve (AUC) of 0.83 for the detection of significant coronary artery disease during exercise stress testing. This finding demonstrates how AI can bridge diagnostic and rehabilitative applications, supporting better patient stratification and intervention planning.

Conceptual contributions included Meder et al. [10] emphasizing population-level applications for preventive strategies and scalable rehabilitation.

Taken together, these outcomes reflect AI’s capacity to generate improvements across multiple domains, including behavioral adherence, physiological fitness, cardiovascular risk factors, diagnostic accuracy, and healthcare system scalability. While preliminary, these findings collectively demonstrate AI’s promise as a transformative tool in cardiovascular rehabilitation.

## 4. Discussion

This scoping review reveals that, although the field is still developing, artificial intelligence (AI) has already demonstrated multiple pathways through which it can enhance exercise-based cardiovascular interventions. The most prominent contributions include personalizing exercise prescriptions, adaptive goal setting, real-time monitoring via wearable devises, and utilizing digital engagement strategies to enhance adherence. Collectively, these advances represent an early but tangible shift from standardized rehabilitation protocols toward more dynamic and individualised care.

### 4.1. Interpretation of Findings 

The earliest exploratory studies in this area, notably those by Kwon et al. [6] and Piwek et al. [7], provided the first indications that AI techniques could be meaningfully integrated into exercise-based cardiovascular care. Kwon and colleagues demonstrated how machine learning algorithms, when paired with wearable sensors, can generate exercise prescriptions that adapt to the individual’s physiological responses rather than relying on generic recommendations [6]. Although limited in scale and duration, this pilot study was instrumental in showing that exercise guidance could be personalized in a data-driven manner, moving beyond traditional standardized protocols. In parallel, Piwek and collaborators examined the emerging role of consumer-grade wearable technologies [7]. They highlighted both the promise and limitations of such devices: on one hand, the ability to continuously capture physical activity data in free-living environments. At the same time, they noted that early consumer devices set the stage for more advanced AI-driven monitoring systems, which could bridge gaps between clinical assessments and daily life.These early works, while not definitive, served as proof-of-concept studies that opened the door for subsequent, more rigorous trials.

The first stronger evidence base was established in 2018. Zhou et al. [8] conducted one of the earliest randomized controlled trials in this field and demonstrated that adaptive step goals, generated using machine learning, could substantially increase daily physical activity compared with fixed, non-individualized prescriptions. This was a pivotal finding because it showed that adaptive algorithms could dynamically adjust targets in response to real-time performance, thereby maintaining motivation and engagement more effectively.Around the same time, Aguilera et al. [9] tested a reinforcement learning–based system that delivered personalized text messages to individuals with diabetes and depression. Their trial revealed not only improved adherence but also higher daily step counts, emphasizing the capacity of AI to sustain behavioural change in real- time in populations with complex comorbidities, who often present challenges for standard rehabilitation programs. Taken together, these two RCTs represented a turning point, as they provided the first controlled evidence that AI-enhanced personalization could outperform conventional, one-size-fits-all interventions in promoting sustained physical activity.

Subsequent research advanced from feasibility toward clinical relevance. Leitner et al. [16] implemented an autonomous AI health coach capable of delivering personalised lifestyle recommendations and demonstrated significant reductions in blood pressure among adults with hypertension. This scalability suggested AI-driven interventions could reduce clinician burden while maintaining patient outcomes.Meanwhile, Hsiao et al. [17] validated a wearable device enhanced with AI algorithms to estimate oxygen consumption continuously using multispectral photoplethysmography. This bridged the gap between laboratory-based testing and unsupervised rehabilitation, ensuring safer and more personalized training at home.Xiao et al. [15] extended the application of AI into geriatric care, applying a neural network model to design individualized exercise prescriptions for older adults, which resulted in measurable improvements in aerobic capacity and cardiorespiratory fitness. Together, these studies highlight a transition from exploratory proof-of-concept toward clinically meaningful applications, underscoring AI’s dual role in both prevention and rehabilitation.

Other contributions expanded the scope beyond direct exercise prescription or monitoring. Liang et al. [14] applied deep learning methods to exercise stress testing, achieving improved accuracy in detecting significant coronary artery disease. This line of work demonstrates how AI can serve not only as a rehabilitation tool but also as a diagnostic adjunct, thereby strengthening the continuum between disease detection, risk stratification, and therapeutic intervention. Meder et al. [10], in contrast, emphasized a macro-level perspective, examining how AI could contribute to population health through enhanced risk prediction, prevention strategies, and resource allocation. Taken together, these contributions illustrate that AI is not confined to the micro-level of individual patient management but may also serve as a systemic innovation capable of reshaping broader healthcare practices.

### 4.2. Clinical Implications

The findings of this review carry several important implications for clinical practice, particularly in the context of cardiac rehabilitation and preventive cardiology. AI-enabled tools have the capacity to optimise exercise prescriptions by matching training intensity and duration to individual physiological characteristics, thereby improving both safety and effectiveness. For example, machine learning–driven algorithms can analyze variables such as heart rate responses, baseline VO_2_, or comorbid conditions to tailor exercise prescriptions in ways that conventional protocols cannot [6,12]. Such personalization reduces the likelihood of undertraining or overexertion and allows programs to be fine-tuned to maximise clinical benefit.

Another central clinical implication is the ability of AI to enhance adherence, which is often a key challenge for long-term rehabilitation programs. By embedding reinforcement learning principles into digital coaching systems, AI can adjust motivational strategies in real-time, delivering tailored prompts that respond to patient progress and behavioural patterns [9,18,19]. Unlike static programs, these adaptive systems evolve in tandem with the patient, maintaining engagement over extended periods. This adaptability is particularly valuable given that most lifestyle interventions suffer from declining adherence after the first few months, undermining their long-term effectiveness.

The potential of hybrid care models deserves special emphasis. In addition, AI may help overcome geographical and logistical barriers by extending cardiac rehabilitation into home and community settings. This division of labor reduces clinician burden while enhancing patient safety through early detection of abnormal responses. Moreover, AI-enabled wearables and remote monitoring platforms expand rehabilitation beyond hospital walls. This could be transformative in rural and resource-limited regions, where access to specialized centers is scarce [20,21]. By democratizing access to personalized rehabilitation, AI can reduce disparities and make preventive care more equitable.

Another layer of clinical impact lies in the integration of AI-enabled rehabilitation with broader digital health infrastructures. Exercise data collected from wearables and mobile platforms can be incorporated into electronic health records, creating more comprehensive patient profiles. When data from AI-enhanced wearables feed into electronic health records, continuity of care improves, facilitating collaboration among cardiologists, physiotherapists, and primary care providers.

Ultimately, the clinical implications extend to the efficiency of the health system. By automating routine monitoring and patient engagement tasks, AI can reduce the burden on healthcare professionals and potentially lower the costs of rehabilitation programs. In this way, AI strengthens both individual-level precision medicine and population-level preventive strategies

### 4.3. Ethical, Regulatory, and Practical Challenges

The promising results must be tempered by recognition of key challenges. One of the central issues is algorithmic opacity. Many AI models operate as ‘black boxes,’ providing outputs without transparent explanations of the underlying decision-making process [4,5]. This lack of interpretability undermines clinician trust, complicates regulatory approval, and limits patient acceptance. Future research must prioritise explainable AI (XAI) approaches that make algorithmic reasoning accessible and verifiable to healthcare providers. 

Another major challenge is equity and accessibility. AI-driven rehabilitation often depends on smartphones, wearable sensors, and reliable internet connectivity. These technologies, however, are not universally available. Patients in low-resource settings, older adults with limited digital literacy, and disadvantaged groups may be disproportionately excluded. Designing interventions that are both technologically sophisticated and user-friendly, affordable, and adaptable is therefore crucial to prevent preventing the widening of health disparities. 

Privacy and data governance also remain pressing concerns. Continuous monitoring generates sensitive physiological and behavioral data, raising questions of ownership, consent, and secondary use [18]. Robust cybersecurity measures and transparent governance frameworks will be essential to safeguard trust. 

Integration into healthcare systems poses practical obstacles. Clinicians must be trained to interpret AI outputs, while workflows and reimbursement structures need adaptation to accommodate digital health innovations. Without structural adjustments, even well-validated AI solutions may fail in real-world uptake. Importantly, cost-effectiveness analyses are necessary to demonstrate value and support reimbursement decisions. 

In summary, while AI offers significant promise, its clinical translation depends on addressing these ethical, regulatory, and practical challenges. Failure to do so risks undermining both clinician confidence and patient outcomes.

### 4.4. Research Gaps and Future Directions

The current body of evidence on AI-enabled exercise interventions for cardiovascular health is still relatively modest, fragmented, and characterised by considerable methodological heterogeneity. One of the most pressing priorities for the field is the design and implementation of large-scale, multicenter randomised controlled trials (RCTs) involving diverse patient populations [8,9]. To date, only two RCTs have been conducted, both with encouraging results, but small sample sizes and population specificity limit the generalizability of their findings. Broader trials are necessary to validate efficacy across different demographic groups, clinical conditions, and healthcare contexts.

In addition, pragmatic trials embedded within routine clinical workflows will be essential to assess not only efficacy but also cost-effectiveness and feasibility in real-world contexts.characterised by considerable methodological heterogeneity.

Another priority is the establishment of standardised frameworks for validation, reporting, and evaluation. Currently, studies employ highly variable AI algorithms, exercise modalities, and outcome measures, limiting comparability. Consensus guidelines could improve consistency and facilitate meta-analysis in the future.

Transparency and interpretability of AI models also remain significant concerns. Reliance on black-box deep learning approaches reduces clinician confidence. Explainable AI (XAI) tools should be prioritised to ensure outputs align with medical reasoning and can be trusted in clinical decision-making.

Research must also address equity of access. Most existing studies are concentrated in technologically advanced regions [22,23]. Future interventions should deliberately include underrepresented groups and be tested in low-resource settings to avoid widening health disparities.

Finally, long-term follow-up is needed to determine whether short-term improvements in adherence, blood pressure, or VO_2_max translate into reductions in morbidity, mortality, and healthcare utilisation. Opportunities also exist to explore synergies between AI and other digital health innovations, such as telemedicine, gamification, and virtual reality.

## 5. Strengths and Limitations

This review’s strengths include its novelty, systematic methodology, and comprehensive scope. To our knowledge, it is the first synthesis to focus exclusively on AI applications in exercise-based cardiovascular interventions. It adhered to PRISMA-ScR guidelines [13], used multiple databases, and covered a decade of research. A range of study designs was included, offering a broad perspective on the field. In addition, by structuring the review according to the PCC (Population–Concept–Context) framework, the study ensured transparency and reproducibility, which strengthens the credibility of the synthesis. The multidisciplinary nature of the databases searched further reinforces the comprehensiveness of the evidence captured.

Limitations include the small number of eligible studies (*n* = 9) and the predominance of exploratory designs. Only two RCTs were identified [8,9], restricting the strength of conclusions. Considerable heterogeneity in populations, AI methodologies, and outcomes prevented meta-analysis. Publication bias may have inflated positive findings, and most studies were conducted in technologically advanced settings [7,21,24], which limits generalizability. Few addressed ethical issues such as algorithmic transparency, [3,25], data privacy, or equity of access. Furthermore, the limited follow-up duration in most feasibility and pilot studies constrains conclusions about the sustainability of benefits over time. The lack of standardized frameworks for reporting and validating AI interventions further complicates comparisons across studies. Natably, very few investigations included diverse or resource-limited populations, raising concerns about the equity and global applicability of current evidence. These gaps highlight the need for future large-scale, multicenter trials with harmonized methodologies and more extended follow-up periods.

## 6. Conclusions

This scoping review synthesized evidence on AI in exercise-based cardiovascular interventions. Nine studies illustrated AI’s ability to personalize exercise prescriptions, set adaptive activity goals, support continuous monitoring and improve adherence. Diagnostic and generative applications further expanded the scope. Beyond these specific domains, the review highlights the potential of AI to bridge clinical rehabilitation with preventive cardiology, offering scalable solutions that can be adapted to both individualized and population-level strategies. Such versatility underscores AI’s role not only in patient management but also in reshaping healthcare delivery models.

AI has the potential to overcome persistent barriers in rehabilitation and prevention by aligning with the principles of precision medicine However, the evidence remains preliminary. More RCTs, armonized methods, and studies in diverse populations are urgently needed. Ethical and equity concerns, including algorithmic transparency, data protection, and accessibility, must be addressed before large-scale adoption.

If these safeguards are implemented, AI can evolve from experimental applications into validated, evidence-based components of cardiovascular care. Its integration into hybrid care models will allow clinicians to extend supervision beyond hospital walls, improving both access and continuity of care. Ultimately, AI should be regarded as a complementary tool that augments clinical expertise, helping to reduce the global burden of cardiovascular disease through more personalized, efficient, and equitable rehabilitation strategies.

## Data Availability

No new data were created or analyzed in this study. Data sharing is not applicable to this article.

## Supplementary Materials

The following supporting information can be downloaded at: https://www.mdpi.com/article/10.3390/jfmk10040409/s1, Table S1: Database Search Strategies (2015–2025); Table S2: Characteristics of Included Studies (2015–2025); Table S3: Summary of Study Characteristics (*n* = 9).
