# Supplementary material for: The Role of Artificial Intelligence in Exercise-Based Cardiovascular Health Interventions: A Scoping Review
Source: J Funct Morphol Kinesiol. 2025 Oct 21;10(4):409. doi: 10.3390/jfmk10040409 (PMC12550945; doi:10.3390/jfmk10040409)
Supplement: Supplementary file 1 [file jfmk-10-00409-s001.zip › jfmk-3923624-supplementary.pdf]

JFMK-3923624- Supplementary

Table S1. Database Search Strategies (2015–2025).

Database	Search Query	Filters / Notes
PubMed	("artificial intelligence"[MeSH Terms] OR "machine learning"[MeSH Terms] OR "deep learning"[All Fields]) AND ("exercise"[MeSH Terms] OR "physical activity"[All Fields] OR "cardiac rehabilitation"[All Fields]) AND ("cardiovascular diseases"[MeSH Terms])	Humans, 2015–2025, English only
Scopus	TITLE-ABS-KEY("artificial intelligence" OR "machine learning" OR "deep learning") AND TITLE-ABS-KEY("exercise" OR "physical activity" OR "cardiac rehabilitation") AND TITLE-ABS-KEY("cardiovascular")	Document type = Article, 2015–2025, English
Web of Science	TS=("artificial intelligence" OR "machine learning" OR "deep learning") AND TS=("exercise" OR "physical activity" OR "cardiac rehabilitation") AND TS=("cardiovascular")	Timespan: 2015–2025; Document type: Article; English
Embase	('artificial intelligence'/exp OR 'machine learning'/exp OR 'deep learning'/exp) AND ('exercise'/exp OR 'physical activity'/exp OR 'cardiac rehabilitation'/exp) AND ('cardiovascular disease'/exp)	Humans only, 2015–2025, English
IEEE Xplore	("artificial intelligence" OR "machine learning" OR "deep learning") AND ("exercise" OR "physical activity" OR "cardiac rehabilitation") AND ("cardiovascular")	Years: 2015–2025; Document type: Journals/Conferences

Table S2. Characteristics of Included Studies (2015–2025).

Authors (Year)	Journal	Population / Design	AI Application	Exercise Mode	Key CV Outcomes
Kwon J. (2016)	Med Biol Eng Comput	Cardiac rehab; pilot	ML individualised prescription	Supervised aerobic+resistance	Better HR/VO_2 matching, ↑ adherence
Piwek L. (2016)	PLoS Med	Healthy & CVD risk	AI-enhanced wearables	Free-living PA	Improved monitoring, feasibility
Zhou M. (2018)	JMIR Mhealth Uhealth	Adults; RCT	ML adaptive step goals	Daily walking	↑ Steps vs static targets
Leitner J. (2024)	JMIR Cardio	Hypertensive adults; non-RCT	Microguides from BP & wearables	Home PA	↓ BP, ↑ adherence
Aguilera A. (2024)	NPJ Digit Med	At-risk adults; RCT	RL personalised SMS	Walking	↑ Steps, ↑ adherence
Hsiao C-T. (2025)	Sensors	Cardiac rehab; feasibility	ML-PPG VO_2 estimation	Home rehab	Continuous VO_2 monitoring
Xiao Y. (2025)	Front Physiol	Older adults; non-RCT	Neural-network prescription	Aerobic training	↑ VO_2max
Liang H-Y. (2025)	Front Artif Intell	818 patients; DL ExECG	CNN-LSTM	Stress test	↑ CAD diagnostic accuracy
Meder B. (2025)	Eur Heart J	Perspective	AI strategies	Conceptual	Policy framework, Potential personalisation

Abbreviations: AI = Artificial Intelligence; BP = Blood Pressure; CAD = Coronary Artery Disease; CNN-LSTM = Convolutional Neural Network–Long Short-Term Memory; CVD = Cardiovascular Disease; CV = Cardiovascular; DL = Deep Learning; ExECG = Exercise Electrocardiogram; HR = Heart Rate; ML = Machine Learning; ML-PPG = Machine Learning–Photoplethysmography; PA = Physical Activity; RCT = Randomised Controlled Trial; RL = Reinforcement Learning; VO_2 = Oxygen Consumption; VO_2max = Maximal Oxygen Uptake; ↑ = Increase/Improved; ↓ = Decrease.

Table S3. Summary of Study Characteristics (n=9).

Category	Distribution
Study Design	2 Pilot (22%), 2 Feasibility (22%), 2 Non-randomised (22%), 2 RCTs (22%), 1 Perspective (12%)
AI Applications	Adaptive goals (2), Neural-net (1), Stress test DL (1), RL nudges (1), Wearable monitoring (2), Coaching (1), Policy perspective (1)
Exercise Modalities	Aerobic/PA (6, 67%), Combined (1, 11%), Home rehab (1, 11%), Stress test (1, 11%)
Reported Outcomes	BP reduction (2, 22%), Adherence/engagement (3, 33%), ↑ Steps (2, 22%), Continuous VO_2 (1, 11%), ↑ VO_2max (1, 11%), Diagnostic accuracy (1, 11%), Conceptual (1, 11%)

Abbreviations: AI = Artificial Intelligence; BP = Blood Pressure; DL = Deep Learning; PA = Physical Activity; RCT = Randomised Controlled Trial; RL = Reinforcement Learning; VO_2 = Oxygen Consumption; VO_2max = Maximal Oxygen Uptake; ↑ = Increase/Improved.
